# Tissue Engineering and Three-Dimensional Printing in Periodontal Regeneration: A Literature Review

**DOI:** 10.3390/jcm9124008

**Published:** 2020-12-11

**Authors:** Simon Raveau, Fabienne Jordana

**Affiliations:** 1 Dental Faculty, University of Nantes, 44000 Nantes, France; simon.raveau@hotmail.fr; 2 Dentistry Department, University Health Centre, 44000 Nantes, France

**Keywords:** three-dimensional printing, tissue engineering, guided tissue regeneration, periodontal, tissue scaffolds, stem cells, growth factors

## Abstract

The three-dimensional printing of scaffolds is an interesting alternative to the traditional techniques of periodontal regeneration. This technique uses computer assisted design and manufacturing after CT scan. After 3D modelling, individualized scaffolds are printed by extrusion, selective laser sintering, stereolithography, or powder bed inkjet printing. These scaffolds can be made of one or several materials such as natural polymers, synthetic polymers, or bioceramics. They can be monophasic or multiphasic and tend to recreate the architectural structure of the periodontal tissue. In order to enhance the bioactivity and have a higher regeneration, the scaffolds can be embedded with stem cells and/or growth factors. This new technique could enhance a complete periodontal regeneration. This review summarizes the application of 3D printed scaffolds in periodontal regeneration. The process, the materials and designs, the key advantages and prospects of 3D bioprinting are highlighted, providing new ideas for tissue regeneration.

## 1. Introduction

The goal of periodontal therapy is the regeneration of the entire periodontal complex in order to recreate its original architecture and function. This involves bone formation, cement formation on the dental root and the attachment of periodontal fibers between the root surface and the alveolar bone.

Conventional periodontal treatments, surgical or non-surgical, do not allow a complete periodontal regeneration. They can only obtain a partial localized regeneration in the apical part of the bone defect. Non-functional conjunctive fibers form on the rest of the defect creating a junctional epithelium [1].

The healing patterns after a periodontal treatment can be either a regeneration or repair. Concerning the periodontal repair, the migration of epithelial cells in the apical direction will form the long epithelium junction. This long epithelium junctional should be considered a natural event in the healing of the attachment system. It is a restoration of soft tissue continuity. It involves new tissue formation but the architecture and function are not fully restored. A periodontal reattach is therefore formed, which is a simple reunion of gum tissue with the root surfaces following surgical or traumatic separation. Periodontal regeneration is a process considered complex because it requires the entanglement and synchronization of different tissue types; the soft tissues (Sharpey’s fibers and connective tissue) and hard tissues (alveolar bone and cementum) [2]. Several cell types are involved like fibroblasts for the periodontal ligament, cementoblasts to reform the cement and osteoblasts for bone [3]. Tissue regeneration therefore depends on the availability of these necessary cells, the presence of signals capable of controlling their behavior, mechanical tensions and the extracellular matrix (ECM).

On a day to day practice, several techniques and products are actually available. Guided Tissue Regeneration (GTR) is an advancement in the surgical approach for periodontal therapy. This technique involves barrier membranes in order to enhance selective repopulation of the periodontal defect with cells derived from the periodontal ligament [4]. This technique has shown many successful outcomes, but it still suffers from various limitations as well as clinical variability [5]. Indeed, the variability in outcomes is also affected by many local and systemic factors similarly to any material-based approach [6].

Bioactive molecules are also used like amelogenins and growth factors [7,8]. The results are similar to the ones with GTR, but the indications are limited (intraosseous bone defects and class II inter-radiculary lesions) [9,10]. Several papers have reported interesting outcomes in the treatment of intraosseous defects using a combination of amelogenin and bone grafts) [11,12].

Growth factors like bone morphogenetic proteins (BMP-2, BMP-7), the connective tissue growth factor (CTGF), the platelet-derived growth factor (PDGF), the fibroblast growth factor (FGF), the stroll cell-derived factor (SDF1) and amelogenins are incorporated in poly glycolic microspheres which are seeded in the scaffolds in order to enhance periodontal regeneration. These molecules can therefore have a controlled release promoting the recruitment and differentiation of progenitor cells. For example, BMP7 plays a major role in osteoblast differentiation, and thus in alveolar bone mineralization. SDF1 has a chemotactic effect on bone marrow stem cells and endothelial cells, all of which are necessary for angiogenesis. FGF promote fibroblast growth and stimulates the formation of blood vessels. PDGF stimulates cell replication of pre-odontoblasts located inside bone tissue, endothelial cells (neovascularization) and the migration of perivascular cells (macrophages). It has a chemotactic effect on the cells of the periodontal ligament and on the promotion of collagen synthesis. It potentiates other growth factors like IGF-1.

The use of individualized scaffolds could reproduce the hierarchical structure of periodontal tissue and enhance the clinical results of the periodontal regeneration. So the use of three-dimensional (3D) printed scaffolds could be an interesting complementary technique [13,14,15].

The aim of the literature review was to produce an overview of the scope of scaffolds in periodontal regeneration, as well as the different 3D printing techniques and materials used for 3D printing.

## 2. The Different 3D Printing Techniques

### 2.1. Electrospinning

**Figure 1:**
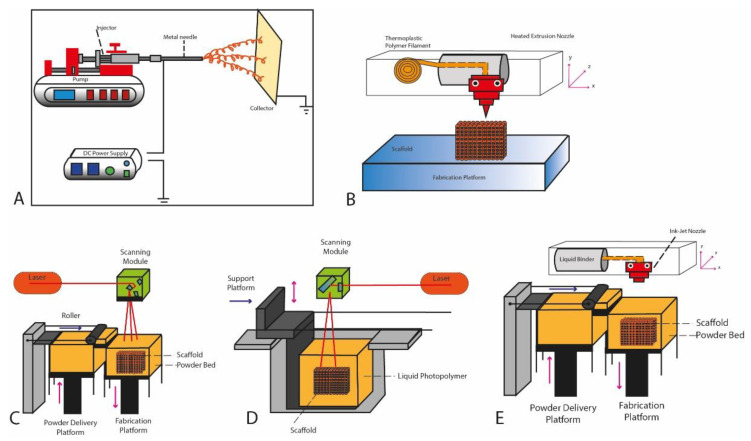
Diagram of the different 3D printing techniques. (**A**) Electrospinning. (**B**) Extrusion. (**C**) Power Bed Fusion. (**D**) Stereolithography. (**E**) Inkjet printing.

Electrospinning is a technique which uses an electric force to draw a charged polymer in order to form a fiber. The system consists of an injection pump, a syringe, a needle, a high voltage power supply and a collector plate. The polymer is pumped by the syringe to the tip of the needle. By applying a high voltage, an electric field is created between the tip of the needle and the collector plate. (Figure 1A) The droplet is distorted forming the so-called Taylor cone. The distortion leads to an electrically charged jet injection that moves towards the collector thus forming thin fibers. The diameter of the needle can be adjusted from nanometers to micrometers. This technique allows control of pore size, porosity, fiber thickness and internal and external geometry [16,17].

Drugs and growth factors can be added to the fibers to endow specific therapeutic properties. For example, a nanofiber-based intracanal drug delivery system has been proposed as a means to create a bacteria-free environment in root canal system of necrotic teeth favorable to tissue regeneration. A polymer solution was loaded with the antibiotics. By adjusting electrospinning parameters (e.g., flow rate, field strength, etc.), antibiotic-eluting nanofibers are obtained [18,19,20].

### 2.2. Material Extrusion

Fused Deposition Modeling (FDM) or Fused Filament Fabrication (FFF) involves heating a high-grade thermoplastic filament, which is extruded through a nozzle to create individual layers (Figure 1B). The collector plate is heated to avoid deformation due to the thermal shock undergone by the material, which passes from more than 200 °C to ambient temperature almost instantaneously [21]. When the material is extruded, the nozzle follows a predefined path determined by the computer in order to form the Computer Aided Design (CAD) model.

Due to the manufacturing process, this technique struggles to create long structures with large overhangs. Extruded filaments generally lack resistance to support themselves immediately after extrusion, resulting in partial or total collapse of the unsupported segment. Therefore, the system is also capable of constructing a supporting structure of a different material which is later discarded by dissolving in water or detergent after the printing [22,23]. However, these materials must be printed simultaneously, which therefore requires printers with two nozzles [24,25].

### 2.3. Powder Bed Fusion

Selective Laser Sintering (SLS) uses a laser and a vat of powder bed. The laser, controlled by the computer, heats the powder just below boiling point (sintering), which fuses the particles in the powder together into a solid form. Once the first layer is formed, the platform of the SLS machine drops, exposing a new layer of powder for the laser to sinter and fuse together. (Figure 1C) This process continues until the entire object has been printed. No supporting structure is needed for this technique since the printed object is encased in powder [26,27,28,29,30].

### 2.4. Stereolithography (SLA)

Stereolithography (SLA) is the first method of 3D-printing which was developed by Charles Hull in 1984. It consists of a vat or tank with photosensitive polymer that is cured by a light source called photo-polymerization. The laser, controlled by a computer-controlled mirror, “draws” the pattern of the object design into the photosensitive polymer. Wherever the laser hits, the liquid solidifies. After the first layer, the platform is raised according to the layer thickness, and additional liquid polymer is allowed to flow below the printed layer. The laser then solidifies the next layer and the process is repeated until the whole scaffold is complete (Figure 1D). SLA technology has improved dramatically with the development of more efficient light sources and improved mirror lens systems, which increase print speed and resolution [31,32,33].

### 2.5. Inkjet Printing

Inkjet printing allows very small volumes (1–100 pL) of individual droplets from a nozzle to be arranged on a printing surface in order to form structures after solidification (Figure 1E). The printing process has been sped up with the development of multi-nozzle head printers with several hundred individual nozzles. Ink jetting process allows the use of several different materials [34,35,36,37].

## 3. The Different Materials Used for 3D Printing 

The properties of scaffolds are influenced by the different biomaterials that constitute them. It is necessary to fully understand the characteristics of each biomaterial in order to choose the one that is optimal for regenerating the desired tissue (Table 1). Indeed, the choice of biomaterials will impact affinity, adhesion, and cell proliferation and thus influence the results of periodontal regeneration.

### 3.1. Natural Polymers 

These are the materials that were used first because they are easy to use and process, and they have a low cost. Regarding their biological properties, they have very good biocompatibility, hydrophilic properties, and good biodegradation. They also allow good cell recognition and improve cell interactions with surrounding tissues. Despite their interesting biological properties, natural polymers lack bioactivity, which is a key factor in promoting the formation of hard tissue. They also have very low mechanical properties and a very high resorption rate by enzymatic reaction. In order to overcome these limitations, the natural polymers constituting the scaffolds are often combined with more bioactive materials (such as bioceramics for example) or mechanically stronger materials (such as synthetic polymers) [38,39,40,41,42,43,44,45].

### 3.2. Synthetic Polymers 

The use of these synthetic polymers is very interesting because they can be produced at low cost, in large quantities and have a longer shelf life than natural polymers. The most used materials are aliphatic polyethers: polycaprolactone, polylactic acid, polyglycolic acid and their co-polymer poly (lactic-co-glycolic) (PLGA). Polycaprolactone is the best known and most widely used aliphatic polyether in the medical field and in particular in craniofacial repair over the past thirty years. It is a material of choice because of its interesting properties. It is biocompatible and usable for many 3D printing techniques. It has a very long resorption time and a high mechanical resistance. Poly-ε-caprolactone (PCL) has a low melting temperature of ~60 °C and rapid solidification due to its semi-crystallinity which make it a good candidate for temperature-based printing techniques. On the other hand, polycaprolactone is hydrophobic, which implies a lower cellular affinity, a decrease in cellular responses and surface interactions. However, in general, aliphatic ethers have a much lower resorption rate than natural polymers and bioceramics. Although they lack bioactivity, aliphatic ethers are interesting because they are very moldable during manufacture and have good mechanical properties [46,47,48,49,50].

Most of the synthetic polymers are hydrophilic. Recently most published papers have performed a specific modification to improve cell–scaffold interaction [51]. Polymers can be biofunctionalized in two different ways. The first one is pre-polymerization functionalization via polymerization of functional monomers (e.g., alcohols, carboxylic acids, amines, and acrylates) [52]. This procedure provides, for example, functional polyesters or polyurethanes with a defined chemical structure that allow for further modification following polymerization [53]. The second strategy is post-polymerization functionalization, which is the modification of the polymer after the polymerization process [54]. Post-polymerization techniques might be specific, targeting functional groups present in the polymer via carbodiimide or UV-initiated radical coupling, or non-specific, using azide- or glutaraldehyde-based couplings. A disadvantage of the non-specific covalent functionalization method is that it may result in the destruction of biomolecule bioactivity and/or can involve side reactions such as hydrolysis, chain-degradation or cross-linking [55].

### 3.3. Bioceramics 

Bioceramics are the material of choice for bone reconstruction due to their unlimited availability, their excellent biocompatibility, their hydrophilic properties and their bioactivity. They are very similar to the inorganic components of bone tissue, are osteoconductive and potentially osteoinductive. Bioceramics with intrinsic osteoinductive properties have the ability to trigger the differentiation of non-differentiated cells towards to osteogenic lineage. Chemical composition, macropore size and geometry, microporosity, surface microstructure and specific surface area have been shown to play key roles in bone induction. The natural ability of calcium phosphate (CaP) to bind BMPs with the presence of concavities within the scaffold that helps the retaining and concentrating BMPs and ions in the vicinity of the scaffold, creating a favorable niche for the differentiation of mesenchymal stem cells (MSCs).

The most documented phosphocalcic bioceramic is hydroxyapatite (HA) because it shares the same biochemical composition as bone tissue, which allows adhesion and proliferation of osteoblasts. Despite this important factor, HA resorbed very slowly in vivo compared to other bio ceramics. The second bioceramic the most studied is the tricalcium phosphate *β β* (*β*-TCP), because it induces the formation of a very strong bond between bone and calcium phosphate, and its rate of resorption is significantly higher. The combination of HA and *β*-TCP produces a two-phase ceramic (BCP). It has highly interesting properties such as the control of its bioactivity, a good stability, allows the induction of bone growth especially in very large defects. In addition, its degradation rate can be controlled by the fact that hydroxyapatite has a very low resorption rate (several years) and the *β*-TCP a very high resorption rate (several months). There are also bioactive glasses (BG), made of silicone oxide and substituted calcium [56,57,58,59,60,61]. In contact with biological fluids, a layer of calcium phosphate is formed on the surface of the bioglass which allows chemical bonding with the surrounding bone.

Despite all the qualities of ceramics, they remain very fragile and difficult to model due to their rigidity, their elastic moduli and their low wettability. They have low mechanical strength and low fracture resistance. However, it is possible to reduce their fragility, their difficulty in being shaped and their low mechanical resistance by combining them with other materials such as polyethers.

Each material has its own characteristics and individual limitations. These biomaterials are often combined in order to have a synergistic action combining all the mechanical and biological properties of materials in order to increase the mechanical, biological property and the kinetics of degradation of a scaffold.

## 4. Scaffold Structure Necessary for Bone Regeneration

The role of a scaffold is to serve as a support matrix. The disadvantages of allografts, xenografts and alloplastic materials are that they are relatively fragile, their porosity difficult to modify and they are difficult to adapt to the specific needs of the patient. They are unable to maintain the desired volume under mechanical forces, which hinders cell colonization [62]. Autografts are relatively resistant to mechanical stress; it is very difficult to shape their shape so that they adapt perfectly to the patient’s bone defects [63].

Tissue engineering makes it possible to create three-dimensional scaffolds that adapt perfectly to the size and form of the patient’s bone defect in order to optimize cell adhesion, proliferation, differentiation and thus tissue regeneration. The purpose of this technique is to replace the defect by a healthy, functional tissue which corresponds to the original tissue.

In general, scaffolds are hydrophilic and have a specific surface topography [64,65,66]. They must have a specific micro and macro structure in order to reproduce the process of bone formation [67]. Nano-topography increases the available surface and thus the surface/volume ratio and the ruggedness which allows an optimal adhesion between the osteoblasts and the surface of the scaffold [68]. As far as micro-topography is concerned, it facilitates cell penetration, vascularization and the diffusion of nutrients [69]. It offers better spatial organization in order to optimize cell growth and the production of extracellular matrix [70].

Other important criteria are porosity, pore size and interconnectivity. Human cancellous bone has a 30–90% porosity [71]. Too large porosities can jeopardize the mechanical stability of the scaffold by reducing its resistance to compression [72]. For the regeneration of the alveolar bone, the conventional porosity of 70% is often chosen and used in the pre-clinical and clinical studies.

Various methods have been developed to create the porosity, the micro-topography and nano-topography of a scaffold [73,74,75].

A common approach to increase the porosity of electrospun fibrous scaffolds is to incorporate sacrificial structures, such as salt grains or other pyrogens. These structures can easily be removed at a later stage. However, the removal of these sacrificial structure is often followed by pore collapse [76]. Other techniques exist like multilayering of fibers [77], tailoring of the fiber diameter [78], incorporation of sacrificial fibers [79], and post-processing by laser ablation [80]. Traditionally, structures with microwell arrays have been produced by polymer molding [81]. Despite several attempts to fabricate microwells by electrospinning on templates consisting of metal spheres, controlling the size and shape of microwells has been problematic. 

Polymer fiber electrospinning onto special templates has been proven to be an effective method of fabricating fibrous constructs with defined fiber organization [82,83,84,85,86,87]. Templates with two-dimensional (2D) and three-dimensional (3D) micropatterns produced from surface-machined metal and ice substrates [84,85,86,87] or metal wire networks [82,86] have been used to control the fiber density and alignment in constructs. However, these methods are slow, costly, difficult to control, and do not yield a wide range of scalable pattern geometries. Polydimethylsiloxane (PDMS) templates with surface micropatterns produced by photolithography have been used to overcome these drawbacks [88], but with limited success in controlling the fiber density and orientation. 

Silicon wet etching and PDMS molding techniques can be combined to construct micropatterned templates, which can be subsequently used to fabricate fibrous Poly-L-lactide (PLLA) scaffolds by electrospinning. This technique can create fibrous structures with different characteristics, including fiber alignment, locally high/low porosity (density), and microwells of different dimensions for a variety of biological applications. The fabricated micropatterned fibrous scaffolds were shown to significantly affect the cell morphology and enhance cell migration in vitro and cell infiltration in vivo.

The degradation profile must also be taken into account. It is directly dependent on the material(s) used. The rate of degradation and the porosity are correlated. If a scaffold has a rapid degradation rate, it must have a low porosity. Otherwise, a high porosity could compromise its mechanical stability as well as its structural integrity before it is replaced by newly formed bone tissue. Conversely, a scaffold with a low degradation rate can have a high porosity because the large contact surface with the native tissue will accelerate its degradation by macrophages by oxidation and/or hydrolysis [89,90]. 

The implantation of a scaffold triggers a phased wound healing process. The first step is an infiltration of immune cells. Then, tissue producing cells are attracted and is followed by the secretion of extracellular matrix (ECM) components and, ultimately, the regeneration of a functional, organized native-like tissue [91]. The resorption of the scaffold is due to the immune cells infiltrating the scaffold, particularly, to the phagocytes, e.g., neutrophils and macrophages [92,93]. Phagocytes adhere to the scaffold and synthesize large amounts of degradative products, such as hydrolytic enzymes, like lysosomal acid lipase (LIPA) and cholesterol esterase, and/or reactive oxygen species (ROS), a process mediated by the nicotinamide adenine dinucleotise phosphate (NADPH) oxidase-2 complex [94,95,96,97,98]. Neutrophils are responsible for the initial acute inflammatory response. Then macrophages quickly become the predominant cell type and remain present at the biomaterial interface until the degradation process is finalized [99,100]. Scaffold microarchitecture profoundly influences macrophage adhesion, infiltration and differentiation into the classical pro-inflammatory phenotype (M1) and the alternative pro-regenerative phenotypes (e.g., M2a and M2c) [91,101,102,103,104,105,106,107]. Increasing the fiber diameter in the micrometer range improves the expression of M2 markers in vitro [104,107], and the regenerative outcomes in vivo [107]. 

Scaffold resorption mechanisms are different depending on the materials. Polylactides and polyglycolides are hydrolyzed in aqueous media. Some polymers, such as collagen, fibrin and hyaluronan, are decomposed enzymatically via the action of specific enzymes called collagenase, plasmin and hyaluronidase, respectively. Other materials, such as *β*-TCP are resorbed by the action of osteoclasts. These cells are able to release small amounts of hydrochloric acid at the material surface, hence provoking a local change of pH and calcium phosphate dissolution. 

The exact mechanisms explaining these microarchitecture-induced variations in macrophage polarization state are still unclear, but they may be caused by modulation of cellular morphology. 

Induction of a more elongated spindle-shaped morphology using 2D micropatterned substrates in vitro was proposed to stimulate alternative macrophage polarization (M2a) following a distinct actin-related pathway, independent of the biochemical environment [105]. Correspondingly, fiber alignment was shown to minimize the host response, enhancing scaffold–tissue integration while minimizing fibrous capsule formation in vivo [108].

Regarding the diameter of the porosities, 150 and 500 μm allow good vascularization and cellular penetration, without compromising the mechanical stability of the scaffold [50,109]. It is also necessary to have an interconnected porous network in order to allow cell growth inside the scaffold in order to prevent necrosis [47,110]. Another property necessary for the scaffold is its mechanical resistance in order to support colonization, cell differentiation and the growth of neo-tissues. Its mechanical resistance should be very close to the surrounding tissues [111]. In addition, the resorption rate must be adapted to the process of formation of the adjacent tissues. Regarding the alveolar bone, it is considered that the degradation takes place in 5 to 6 months [112]. 

The scaffold must also be biocompatible and bioactive; it should not cause an inflammatory or cytotoxic reaction [113]. The scaffold must induce a specific biological response which leads to bone formation [114]. 

## 5. Computer Assisted Design and Manufacturing 

Computer Aided Design and Manufacturing can be used in order to create a scaffold. 

The first step is the using a Computed tomography (CT) scan or cone beam computed tomography (CBCT) of the defect. The next step is to create a Computer Aided Design (CAD) of the individualized scaffold specific to the defect of the patient. This is followed by exporting the CAD file to a generic 3D file, usually STereoLithigraphy (stl) file format [115]. The stl file is then output to the 3D printed software for converting or slicing into G-code, which is a numerical control programming language used by computer-aided manufacturing (CAM)-automated 3D printers. After loading the material of choice into the printer and selecting the printing parameters, the G-code is initiated and the scaffold is printed. After the scaffold is printed, the final stage is post-processing in preparation for use. Post processing depends on the type of 3D printing process and may involve trimming supports, removing excess powder, or dissolving or rinsing the supporting structures. Depending on the material and the printing technique used, the scaffold may require additional processing like UV curing, thermal treatment, or sintering.

The scaffolds manufactured by 3D printing have shown promising results of periodontal regeneration in preclinical studies. There is therefore a need for further evaluation for future use of this technique in clinical practice [116].

## 6. Applications of Tissue Engineering and 3D Printing for Periodontal Regeneration 

### 6.1. Tissue Engineering in Periodontal Regeneration 

The periodontal ligament is a vascularized and innervated connective tissue composed of highly organized collagen fibers (type I and III). The periodontal ligament has fibers oriented perpendicularly to the cementum and the alveolar bone. Their endings (Sharpey fibers) attach in these mineralized structures in order to stabilize the dental root, transmit occlusal forces and ensure a sensitive function. The presence of cementum and alveolar bone is essential for the formation of a functional periodontal ligament, and for the complete regeneration of the periodontal tissue [1].

Conventional periodontal techniques, surgical and non-surgical, do not reconstruct the structure and initial function of this periodontal tissue [1].

Periodontal healing occurs by the formation of a long junctional epithelium attached to the tooth surface, without ad integrum regeneration of the periodontal ligament, cementum or surrounding bone. This has therefore led to the development of surgical techniques intended to achieve a complete periodontal regeneration in a reliable and predictable manner.

The most widely used approach uses the principle of GTR. The concept of GTR is based on the fact that periodontal tissues have different biological growth speed. The proliferation speed of epithelial cells is higher than bone cells thus causing the filling of the available space by the non-mineralized epithelial and connective tissue. The establishment of barrier membranes promotes selective cell repopulation of the periodontal defect by preventing the proliferation of epithelial cells in favor of the bone and periodontal ligament cells [4].

The use of bioactive molecules and growth factors, such as amelogenins, fibroblast growth factors (bFGF, FGF-2), platelet-derived growth factors (PDGF), platelet-rich plasma (PRP), Bone Morphogenetic Proteins (BMP) have also been used. These molecules regulate the migration, differentiation, proliferation of periodontal and mesenchymal stem cells, as well as their chemotaxis and the production of cell-specific extracellular matrix [10,117].

However, the results concerning the use of barrier membranes and growth factors remain unpredictable, and periodontal regeneration is incomplete [118]. The major limitation remains the inability to exercise space-temporal control over the healing process. Recently, tissue engineering techniques have been developed to overcome this limitation [2].

The basic concept of periodontal tissue engineering is to combine a scaffold with living cells and/or biologically active molecules to form a “Tissue Engineered Construct” (TEC). This scaffold, with an adequate blood supply, will promote tissue regeneration [2,119].

To date, most approaches to periodontal tissue engineering have focused on the use of stem cells to promote a new periodontal attachment. Periodontal ligament stem cells, as well as mesenchymal stem cells, have been used with promising results [120]. Stem cells often need a vector like a scaffold, which will then be implanted in the periodontal defect. The disadvantage of this approach is the inability to deliver these cells to specific locations in the periodontal tissue. The use of cell sheets allows cells to be delivered in a more controlled and targeted manner in the periodontal defect [121].

Cell sheets are a recent technology using temperature-sensitive cell culture dishes that reversibly respond to temperature changes [122]. Periodontal ligament cells cultivated using this technique have a promising regenerative potential after transplantation into different animal models [123,124,125,126,127]. One of the main problems with the use of cell sheets is the difficulty in achieving biomechanical fixation.

**Figure 2:**
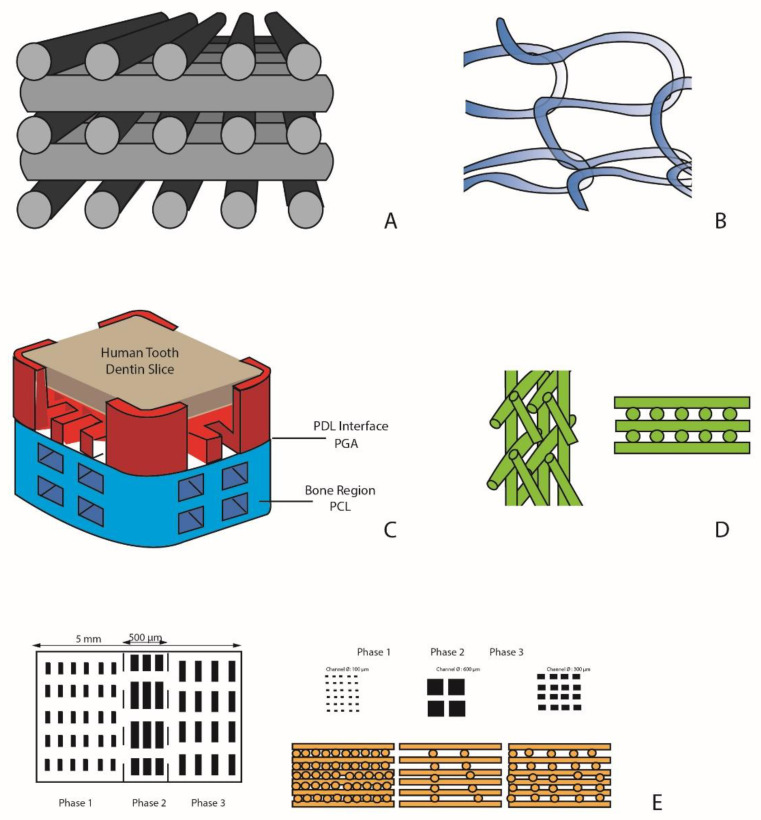
Scaffold designs. (**A**) porous block of laminated strands of biphasic ceramic (α-tricalcium phosphate (TCP) + hydroxyapatite (HA)) [132]; (**B**) monophasic scaffold of poly-L-lactic acid mesh associated with Bone Marrow-derived Mesenchymal Stem Cells with platelet-rich plasma (PRP) [131]; (**C**) biphasic scaffold with two compartments: a bone compartment (poly-ε-caprolactone PCL) and a ligament compartment (acide polyglycolique PGA) [135]; (**D**) biphasic scaffold with a bone compartment and a ligament compartment [89]; (**E**) three-phase scaffold with distinct phases corresponding to the morphology of the periodontal complex: cement, periodontal ligament, and alveolar bone [140].

Thus, the use of a scaffold can provide mechanical support for the periodontal cell sheets and it could also create and maintain the space necessary for bone formation in the periodontal defect. The use of scaffold would override many of the limitations of current clinical practice. They will have the ability to guide and coordinate the healing process. These scaffolds can have one or several compartments and be used alone or in combination with bioactive molecules, medicines, and gene therapy and/or cell delivery (Table S1 [128,129,130,131,132,133,134,135,136,137,138,139,140,141,142,143,144], Figure 2).

### 6.2. Monophasic Scaffolds

The first scaffolds to be designed have only one compartment. They meet the requirements of guided tissue regeneration: wound stabilization, selective cell repopulation, while allowing spatio-temporal control of the periodontal healing process. They can be loaded with cells or growth factors to enhance and promote bone and/or ligament formation.

#### 6.2.1. Simple Monophasic Scaffolds

The Osteoflux, developed by Carrel et al. [132], is a block of laminated strands of biphasic ceramic (α-TCP + HA) printed in 3D by extrusion. It is composed of orthogonal layers of cylindrical filaments. This scaffold was implanted in sheep calvaria and compared to bovine bone (Bio-oss) and *β*-TCP particles to assess vertical bone regeneration. Particulate materials and the 3D block differ in two ways. First, the particulate aspect prevents large volume reconstructions. The use of 3D printed blocks allows both horizontal and vertical augmentation, while the simple use of granules allows only horizontal augmentation. Second, when using particles, the practitioner has no control over the arrangement of pores and inter-particle channels; it is random. This lack of structural organization can limit osteoconduction. One of the advantages of 3D blocks is their structure of linear pores controllable in size and permeability over the entire length of the block. Such a structural organization can promote the progression of the mineralization front with its vascular system. Indeed, 3D printing techniques by extrusion allow to the creation of a controlled and reproducible architecture with 60% of total porosity, channels of 250 µm in diameter with an inter pore distance between 150 and 500 µm thus promoting osteoconduction. The authors were thus able to observe a significant increase in bone growth during the first two months; then at 4 months, there was no significant difference between the materials.

Another type of monophasic scaffold was manufactured by extrusion, by Mangano et al. [129]. It was composed by 30% HA, 60% *β*-TCP and 10% α-TCP. It has a characteristic mesh-like structure with rod diameters of 300 ± 30 μm, and pore sizes between the rods of about 370 ± 25 μm. Its macroporosity is 60%. This scaffold was implanted in a sheep sinus. At 45 days, the authors observed good immuno-tolerance of the scaffold, as well as complete tissue integration, and bone remodeling located at the periphery. At 90 days, they observed the formation of a mineralized lamellar bone at the periphery. In the center, a highly vascularized fibrous tissue formed, showing some fibroblasts and a large vascular network comprising capillaries and large vessels with great structural organization. Beyond 90 days, the scaffold continued its gradual resorption. However, it was not entirely replaced by the newly formed bone tissue (formation of fibrous tissue in certain areas).

#### 6.2.2. Single-Phase Scaffold for Cell Delivery

This technique increases the healing potential by seeding different cell types in the structure. This approach is well documented in the literature. The cells are encapsulated in hydrogels or seeded directly in the scaffolds, which are then implanted in bone defects. Thus, in addition to their role of space maintainer, TECs allow the diffusion of cells in the periodontal defect. While the concept may seem relatively simple, its implementation has led to variable results depending on the biomaterials and the types of cells used.

Baba et al. [131] created by electrospinning a poly-L-lactic acid mesh associated with Bone Marrow-derived Mesenchymal Stem Cells with PRP. In a phase 1 and 2 clinical trial, these scaffolds were implanted in human periodontal defects. They were able to observe a gain in clinical attachment, bone growth and a reduction in pocket depth.

Dan et al. [138] manufactured by electrospinning a CaP-coated PCL scaffold associated with a PCL cell sheet obtained from culture of gum marginal cells, periodontal ligament cells and alveolar bone cells. This scaffold was implanted in rat periodontal defects created surgically.

Bone and ligament growth were observed. All three cell types have shown potential for mineralization. In vivo, only alveolar bone cells and periodontal ligament cells were able to obtain periodontal ligament formation after 4 weeks. The gum marginal cells in particular have shown properties close to those of mesenchymal stem cells.

Cell-based periodontal therapies have considerable regulatory limitations related to cell source, collection and culture. However, the development of advanced scaffolds made of synthetic biomaterials loaded with drugs and/or growth factors makes it possible to find interesting clinical alternatives without a cellular component.

#### 6.2.3. Monophasic Scaffolds for the Release of Growth Factors

For the delivery of growth factors, synthetic polymers are used with a delayed degradation profile and improved mechanical properties. The direct incorporation of biological elements into the structure of the scaffold is complicated by the high temperature manufacturing process as well as the use of strong organic solvents resulting in the denaturation of these biological elements.

The development of microspheres as vectors has made it possible to overcome these limitations and are now widely used in tissue engineering and in particular in periodontal regeneration.

Cho et al. [133] designed a PCL scaffold printed by extrusion, loaded with PLGA microspheres containing BMP-2, BMP-7 and connective tissue growth factor. This scaffold was implanted in vitro on the root surface of human teeth. The incorporation of the microspheres in the TEC radically modifies the release profile of the encapsulated molecules which then reaches a release of 50% after 42 days. Only BMP-7 induces the formation of a cementoid tissue deposit. However, the delayed release of growth factors induces cementogenesis in a later phase which could compromise the insertion of periodontal ligament fibers on the root surface.

Kim et al. [128] designed a scaffold of PCL + HA printed by extrusion, associated with a mixture of SDF1 and BMP-7. It is a scaffold in the form of a rat molar and a rat incisor with interconnecting micro channels of 200 µm in diameter infused in a mixture of SFD1 and BMP7 (100 ng/mL each) associated with a type 1 collagen solution. At 9 weeks, regeneration of the periodontal ligament is observed as well as a new bone formation at the level of the interface with the scaffold in the form of a rat incisor. The use of SDF1 (stromal cell-derived factor 1) and BMP7 made it possible to significantly recruit more endogenous cells, such as mesenchymal and endothelial stem cells, and to increase angiogenesis compared to the control scaffold without growth factor. Indeed, SDF1 and BMP7 makes it possible to recruit several cell lines. SDF1 has a chemotactic effect on stem cells from bone marrow and endothelial cells, all of which are necessary for angiogenesis. SDF1 binds with chemokine CXCR4, a receptor for these two cell types. BMP7 plays a major role in osteoblastic differentiation, and thus in the mineralization of the alveolar bone. This study once again highlights the value of cell recruitment. This technique would allow easier implementation in clinical practice than cell seeding. In addition, this type of technique has a much lower financial cost.

Carrel et al. [132] associated 100 μg of BMP-2 with Osteoflux and the scaffold was implanted in a sheep. The rate of vertical bone growth, bone maturation and bioresorption of the material almost doubled after implantation in sheep. The resorption rate is well adapted which gives the bone enough time to grow and mature until it acquires its structure. In parallel, the biomaterial was absorbed via cell and chemical mediated processes.

The studies discussed above have focused on the association of growth factors with 3D printed scaffolds.

Mathew et al. [139] were interested in preventing graft exposure and thus bacterial contamination due to contact with intraoral fluids. They developed a membrane produced by electrospinning loaded with antibiotics. Azithromycin was deposited on the scaffold fibers, resulting in release over several weeks. These membranes were then tested in rat calvaria defects. The immunomodulatory properties of azithromycin have led to increased bone formation. Thus, this technique would potentially improve both tissue regeneration (immunomodulatory properties) and protect against bacterial contamination (antibacterial properties).

In the periodontium, the regeneration of the alveolar bone is associated with the regeneration of two other important structures that are the periodontal ligament and the cementum. Thus, in order to have a multi-tissue regeneration, the original concept of the monophasic scaffold has evolved into a polyphasic scaffold. The spatial structure is different in that it has architectural and chemical properties which are closest to the organization of the original tissues [145]. It is therefore necessary to “compartmentalize” in order to produce the spatio–temporal kinetics necessary for the regeneration of the periodontium; alveolar bone on the one hand, and the functional orientation of the periodontal fibers on the other.

### 6.3. Biphasic Scaffolds

Park et al. [135] designed a scaffold with two compartments: a bone compartment and a ligament compartment. The compartments were not directly 3D printed. Wax molds were made by extrusion, and the materials were then casted into these molds. The bone compartment was seeded with periodontal ligament cells transduced by Ad-CMV-BMP7. The structure of the ligament compartment is interesting. It is composed of three superimposed cylinders and is seeded with periodontal ligament cells. This biphasic scaffold was implanted in mice periodontal defects surgically created. This scaffold with controlled architecture allowed a biomimetic multi-tissue compartmentalized formation. Periodontal fibers are formed with an angular orientation to the cementum layer, thus approaching the organization of the native ligament. However, it turns out that the control of cellular directionality in vivo remains unpredictable.

Vaquette et al. [89] designed a scaffold with both a bone compartment and a ligament compartment. The bone compartment is manufactured by FDM in *β*-TCP/PC and it is seeded with osteoblasts. The periodontal ligament compartment is a cell sheet produced by electrospinning. This scaffold is implanted subcutaneously on a slice of dentin in an athymic mouse. They observe bone neoformation, ligament and cement regeneration (but with non-functional periodontal fibers not oriented perpendicularly). The presence of the cell sheet is essential for the formation of cementum on the dentin surface. However, the partially occlusive nature of the membrane is a limitation because it would impede the integration of the neo-formed periodontal ligament in the bone tissue [146].

Costa et al. [90] inspired from Vaquette’s concept and made several changes to it. The bone compartment of *β*-TCP/PCL is coated with CaP and seeded with osteoblasts and the pore size is increased. The ligament compartment is modified by the addition of concentric superimposed rings in the cell sheet made by melt electrospinning allowing the membrane to become permeable to cells. This scaffold was also implanted subcutaneously on a slice of dentin in an athymic mouse. These changes resulted in better bone formation, better oblique orientation of the periodontal fibers (but poorly controlled), and increased vascularity.

The use of biphasic scaffolds has therefore facilitated alveolar and periodontal ligament regeneration. However, the deposit of a newly formed cement remains complicated. It depends on the implantation of differentiated cells in vitro or on the ability of endogenous cells to promote cemental apposition on the root surface. Therefore, recent studies have begun to address the incorporation of a third compartment in order to actively promote cementogenesis.

### 6.4. Triphasic Scaffolds

According to this concept, Lee et al. have developed a three-phase scaffold with a precise architecture and a biochemical gradient [140]. This scaffold is made up of three distinct phases corresponding to the morphology of the periodontal complex: cement, periodontal ligament, and alveolar bone. Each layer has a specific architecture with variable pore sizes (100, 600 and 300 μm). The cementum, periodontal and bone compartments, respectively, thus creating a hierarchical structure. These parameters are chosen according to physiological microbiological characteristics and are also based on their latest studies on the regeneration of soft and hard tissues. In addition to the architectural stratification, a biochemical gradient was added in the various compartments thanks to the incorporation of polyglycolic microspheres charged with growth factors specific to the regeneration of each tissue type. Amelogenin, connective tissue growth factors and BMP-2 were incorporated into the cement, ligament and bone compartments, respectively. These growth factors could therefore produce a controlled release promoting the recruitment and differentiation of progenitor cells. The production of the scaffold was digitally controlled by Computer Aided Design (CAD). On the other hand, the incorporation of the growth factors was carried out manually by loading the microspheres into the specific compartments using pipettes, thus inducing structural variations depending on the different batches. Discontinuous cementogenesis was observed, while notable osteogenesis was observed in the bone compartment. Connective tissue was found interposed between these two mineralized formations with an alignment of the fibers and a ligament attachment on the newly formed cementoid tissue.

The use of a three-phase scaffold for periodontal regeneration is relatively recent and remains largely unexplored because the clinical implementation of this approach is difficult. The complexity of periodontal regeneration lies in its spatial–temporal coordination and the difficulty of reproducing it. The main challenge lies in the formation and integration of a cementum layer on the root dentin surface.

## 7. Limitations and Future Perspective

Aside from these promising possibilities of bone scaffold 3D printing, there are still challenges and limiting factors. The aim of a scaffold is to temporarily mimic the structural and mechanical properties of the natural extra cellular matrix of bone tissue. The limitations to date remain low resolution and often insufficient mechanical properties. From a biological point of view, cell seeding in scaffolds poses the problem of long-term cell viability in these structures. New approaches are developing, such as intrinsic vascularization, which means that vascular induction takes place from the scaffold nucleus to the periphery thanks to the presence of a bioactive matrix and vessels inducing angiogenesis [147,148]. This objective can be achieved with inorganic (copper II) or organic angiogenic factors (for example vascular endothelial growth factors) deposited specifically at the end of a closed pore [149,150]. Both approaches show very good potential for animal model neovascularization and will improve future research on 3D printed scaffold engineering.

From an engineering aspect, the composite approach seems the most promising for bone regeneration with less fragile and brittle ceramic scaffolds, capable of supporting loading and transmitting physiological stress to the cells of surrounding tissues. New, more bioinspired materials are being developed in order to obtain more ductile composite ceramic scaffolds. Certain highly organized structures such as the teeth or pearly layers of mollusk shells reveal mechanical properties which have not yet been achieved in scaffold engineering. Some research is moving in this direction and developing materials with local variations in composition [151,152].

Another key factor in achieving this goal is spatial resolution, allowing for a high level of detail. This implies having fine powder particles (to have thinner layers) with great fluidity. It seems complicated for polymers; because of their ductile behavior, it is difficult to grind the polymers with a reasonable yield. Although ceramic powders are available with desirable particle sizes, there is still a systematic lack of knowledge of the optimal particle size and geometry for 3D printing.

The treatment of powder particles with plasma can improve the fluidity of fine particles. This therefore opens the field to the development of even finer powder particles and thus an increase in the level of resolution which is currently not achieved in traditional 3D printing [152].

Apart from the characteristics of the powder, the high resolution is mainly determined by the size of the projection of the binder. In the past, the Solid Freeform Fabrication remained limited to cutting-edge research, while today, printing technology is democratizing and starting to spread massively. This leads to technical innovations in terms of 3D printing such as the development of new faster and more reliable print heads. Today, the printheads found on the market can go as far as specifying drops of the order of a few picometers. All these innovations will open up new research opportunities in the field of 3D printing for scaffold engineering.

The development of “multi-component” printing will allow the simultaneous printing of different materials associated with multiple cell types and a point-by-point control of bioactive agents.

Future improvements may emerge with regard to accuracy and precision of printing, development of imagery and statistical evaluation of accuracy and reproducibility.

The development of high-resolution CT scanners will allow local quantitative analysis of scaffolds and monitoring of the mineralization process. In order to better understand the micro and macro-mechanical properties, techniques for evaluating failures by imagery will provide new knowledge on the mechanical behavior of these materials under stress.

## 8. Conclusions

The scope of 3D printing and bio-printing in regenerative medicine is wide. It includes tissue engineering of bone, cartilage, skin, vessels, tissue, heart valves and liver tissue, but also applications against cancer for example. Given the high potential of this technology, more and more research laboratories are involved in 3D printing. The number of articles referring to it quadrupled from 2012 to 2018. New journals are introduced, and emerging books are being published. Equipment is becoming more available and affordable, with the emergence of bioprinting companies introducing new printers to the market.

The 3D printing technique is a very versatile method allowing almost unlimited designs and with a very wide variety of materials suitable for scaffold engineering. In periodontal tissue engineering, conventional surgical and non-surgical periodontal techniques do not reconstruct the structure and initial function of the periodontium. Thus, 3D printing of scaffolds is seen as an interesting alternative to these techniques in order to achieve more reliable and predictable periodontal regeneration.

These scaffolds or Tissue Engineering Constructs (TECs) meet the requirements of guided tissue regeneration: space maintenance, wound stabilization, selective cell repopulation, while allowing spatio–temporal control of the periodontal healing process. The use of TECs would bypass many limitations of current clinical practice. They have the ability to guide and coordinate the healing process. They can have one or more compartments and can be used alone or in combination with bioactive molecules (such as growth factors), drugs, and gene therapy and/or cell delivery. A multiphasic TEC generally has several compartments that can be distinguished by their different architecture (porosity, size and shape of the pores, etc.) and/or their different biochemical composition. Using scaffolds for periodontal regeneration is relatively recent (five to ten years) and remains largely unexplored because the clinical implementation of this approach is difficult. In addition, most of the studies are still pre-clinical studies. It is therefore necessary to carry out clinical studies with humans in order to confirm these encouraging results.

## Figures and Tables

**Table 1:** Advantages and disadvantages of the different materials used for periodontal regeneration.

Materials	Advantages	Disadvantages
Natural materials	Good biocompatibility and cellular affinity	Significant degradation rate Weak mechanical properties
Synthetic materials	Good physicochemical and mechanical properties High variability in degradation rate and resorption kinetics	Low bioactivity
Ceramics	Composition similar to bone tissue Osteoconductivity Stimulates bone healing	Not compatible with cell encapsulation Fragility Variety of cellular reactions according to their surface properties

## Supplementary Materials

The following are available online at https://www.mdpi.com/2077-0383/9/12/4008/s1, Table S1: 3D-printed scaffolds for periodontal tissue engineering.
