# Supplementary material for: Tissue Engineering and Three-Dimensional Printing in Periodontal Regeneration: A Literature Review
Source: J Clin Med. 2020 Dec 11;9(12):4008. doi: 10.3390/jcm9124008 (PMC7763147; doi:10.3390/jcm9124008)
Supplement: Supplementary file 1 [file jcm-09-04008-s001.pdf]

Table S1. 3D-printed scaffolds for periodontal tissue engineering.

	Authors	3D printing technology	Printed material(s), bioactive factor(s) and / or cells used	Scaffold geometry	Mesh thickness	Porosity
MONOPHASIC	Kim [128]	Extrusion	PCL (80%) + HA (20%) with SDF1 and BMP-7 (100 ng/mL each)	Shape of tooth (rat mandibular central incisor and human mandibular first molar)	200 µm	200 µm diameter interconnecting channels
	Mangano [129]	Extrusion	30% HA, 60% β-TCP, 10% α-TCP	Blocks with a volume of about 0.14 cm³	300 ± 30 µm	370 ± 25 µm<br>Open interconnecting microporosity with pores sizes of about 1 µm<br>Total porosity : 60%
	Baba [130, 131]	Melt spinning	75/25 PLGA+BMMSCs + PRP	1.5 diameter	-	-
	Carrel [132]	Extrusion	α-TCP + HA (calcium-to-phosphate ratio : 1.43)	Hemispheric blocks	400 µm	250 µm<br>Total porosity: 50–65%<br>Macroporosity: 40–50%
	Cho [133]	Extrusion	PCL scaffolds with microspheres of 75/25 PLGA with BMP-2, BMP-7 + CTGF	Rectangular scaffolds (5×3×0.5 mm³)	200 µm	500 µm transverse pores
	Puppi [134]	Extrusion (wet spinning)	PCL fiber construct and a chitosan/poly(γ-glutamic acid) polyelectrolyte complex hydrogel	Rectangular scaffold: 10 × 10 mm, 50 layers	200–300 µm	200–1800 µm
BIPHASIC	Park [135]	3D wax printing system	Bone compartment : 25% PCL with recombinantadenovirus-encodingmurine BMP-7	Rectangular scaffolds: 1.75 × 5.0 × 4.0 mm	-	Windows dimensions: 0.75 × 0.50 × 0.05 mm³
			PDL compartment: 25% PGA	Rectangular scaffolds: 1.5 × 5.0 × 4.0 mm	Diameter: 0.8 mm<br>Height: 0.3 mm	-
	Park [136]	3D wax printing system	Bone compartment: PCL + BMP-7	3 × 2 × 2 mm	-	0.60 × 0.50 mm² window pores to contact residual bone tissue

			PDL compartment: PCL+PDL cells	3 cylindrical-shaped PDL fiber-guiding architectures per layer 0.225 mm diameter, 0.250 mm ligament interface	-	0.175 mm thick interconnective space between PDL and bone regions
	Vaquette [84, 137] Dan [138] Mathew [139]	Electrospinning	Calcium phosphate coated melt electrospun polycaprolactone (CaP-PCL) scaffold	Small pieces (approximately 3 mm × 1.5 mm × 0.5 mm in size)	500 μm	over 150 μm diameter interconnecting pores
			PCL membrane (associated) (can be loaded with azithromycin)	Small pieces (approximately 8 mm × 5 mm × 0.3 mm in size)	300–400 μm with 3 μm diameter fibers	5–10 μm
	Vaquette [89]	Extrusion (FDM)	Bone compartment: PCL + b-tricalcium Phosphate (β-TCP, 20% wt.)	100 × 100 × 2 mm^3 sectioned into 5 × 5 × 2 mm^3	-	interconnectivity, 70% porosity
		Electrospinning	PDL compartment: PCL	cell membrane: 7 × 9 × 0.4 mm^3	-	-
	Costa [90]	FDM	Bone compartment: PCL + β-TCP (20% wt.)	100 × 100 × 2 mm^3 sectioned into 5 × 5 × 2 mm^3	-	interconnectivity, 70% porosity
		Electrospinning	PDL compartment: PCL	Circular membranes with 8 mm diameter	Fiber diameter: 10–15 μm	Interconnected pore<br>Pore size: 100–400 μm
TRIPHASIC	Lee [140]	Extrusion (layer-by-layer deposition)	PCL/HA scaffolds and poly(lactic-co-glycolic acid) microspheres encapsulating factors<br>Bone compartment: +BMP2	5 × 5× 3 mm^3<br>2.25 mm (width)	-	300 mm microchannels
			PDL compartment: + CTGF	0.5 mm (width)	-	600 mm transverse microchannels
			Cementum/dentin interface: +amelogenin	2.25 mm (width)	-	100 mm transverse microchannels
CUSTOM	Rasperini [141]	SLS	PCL and 4% HA	Customized scaffold: patient's defect	Mean strut length: 600 μm for support of extensions in the PDL region	Channel width for PDGF delivery was ≈ 500 μm

ORIENTE	Jiang [142]	Electrospinning	Biodegradable poly PCE copolymer electrospun nanofibrous mats into porous CHI	30 layers of nanofibers embedded within the porous CHI, with thickness ≈ 4 mm	Diameter (nm): - Random fibers: 574.3±218.2 - Aligned fibers: 616.1±213.1 nm	103.38 ± 49.54 µm between layers
	Kim [143]	Electrospinning	PCL/gelatin (Gel) nanofiber with periodontal ligament cells	30 mm × 4 mm × 200 µm	-	-
	Pilipchuk [144]	SLS	Bone compartment: PCL and 4% HA	Idem [130]: 5.1 × 4.1 × 3.2 mm	≈ 0.7 × 0.7 mm	0.7 × 0.7 mm
		Molding	Periodontal ligament: PCL	Idem [130]: 3.6 × 2.8 × 0.4 mm	400 × 250 µm, with/ without grooves	400 µm wide pores

BMMSCs: mesenchymal cells derived from bone marrow, BMP: Bone morphogenetic protein, CHI: chitosan, CTGF: Connective tissue growth factor, FDM: Fused Deposition Modeling, HA: hydroxyapatite, SLS: selective laser sintering, PCE: poly (e-caprolactone)-poly(ethylene glycol), PCL: polycaprolactone, PDGF: Platelet-derived growth factor, PDL: periodontal ligament, PGA: Polyglycolic acid, PLGA: poly-L-lactic acid, PRP: Platelet Rich Plasma, SDF: Stromal-derived factor, SLS: Selective Laser Sintering, TCP: tricalcium phosphate.
